# Image quality for visualization of cracks and fine endodontic structures using 10 CBCT devices with various scanning protocols and artefact conditions

**DOI:** 10.1038/s41598-023-31099-5

**Published:** 2023-03-10

**Authors:** Jáder Camilo Pinto, Karla de Faria Vasconcelos, André Ferreira Leite, Victor Aquino Wanderley, Ruben Pauwels, Matheus L. Oliveira, Reinhilde Jacobs, Mario Tanomaru-Filho

**Affiliations:** 1 Department of Restorative Dentistry, São Paulo State University (UNESP), School of Dentistry, Araraquara, SP Brazil; 2 OMFS IMPATH Research Group, Department of Imaging and Pathology, Faculty of Medicine, KU Leuven and Oral and Maxillofacial Surgery, University Hospitals Leuven, Leuven, Belgium; 3 Department of Dentistry, Faculty of Health Sciences, University of Brasília, Brasília, Brazil; 4 Division of Oral Radiology, Department of Oral Diagnosis, Piracicaba Dental School, University of Campinas, Piracicaba, SP Brazil; 5 Aarhus Institute of Advanced Studies, Aarhus University, Aarhus, Denmark; 6 Department of Mechanical Engineering, Catholic University of Leuven, Leuven, Belgium; 7 Department of Dental Medicine, Karolinska Institutet, Stockholm, Sweden

**Keywords:** Signs and symptoms, Imaging

## Abstract

The aim of this study was to evaluate CBCT exposure protocols and CBCT devices in terms of image quality for the detection of cracks and fine endodontic structures using 3 conditions of metallic artifacts. An anthropomorphic phantom containing teeth with cracks, isthmus, narrow canal, and apical delta was scanned using ten CBCT devices. A reference industrial CT image was used to detect and measure all structures. Three conditions were created: (1) metal-free, (2) ‘endo’ and (3) ‘implant’ with metallic objects placed next to the teeth of interest. For each condition, three protocols were selected: medium field of view (FOV) standard resolution, small FOV standard and high resolution. The results showed that only small FOV high-resolution metal-free images from two devices (A and H) were appropriate to visualize cracks. For fine structure identification, the best result was observed for small FOV high resolution. However, the visualization significantly worsened in the presence of metallic artefacts. The ability of CBCT images for visualizing cracks is restricted to certain CBCT devices. Once metallic artefacts are present, crack detection becomes unlikely. Overall, small FOV high-resolution protocols may allow detection of fine endodontic structures as long as there are no high-dense objects in the region of interest.

## Introduction

Knowledge of root morphology is important for diagnosis, treatment, and prognosis of endodontic therapy. The initial visualization of challenging anatomical structures, as well as the accurate early diagnosis of cracked teeth using cone-beam computed tomography (CBCT) images is of paramount importance for long-term tooth prognosis, due to its direct influence on the endodontic treatment^1^.

The literature is conflicting regarding the sensitivity and specificity of CBCT images for performing endodontic tasks (such as the detection of root fractures and extra canals)^2–6^. The selection of high-resolution protocols on CBCT devices with small field of view (FOV) and voxel sizes has been strongly recommended to visualize particularities of the root canal system and periodontium^7,8^. Conversely, a systematic review did not find any correlation between smaller voxel sizes and better diagnostic accuracy of root fractures in non-endodontically treated teeth^5^. Furthermore, the effect of voxel size may be device-dependent due to other limiting factors for spatial resolution, such as the type of the detector and the reconstruction process^8^. Technical characteristics vary widely among commercially available CBCT devices^10,11^, which makes it difficult to generalize diagnostic capability for all devices and acquisitions protocols^8^. Moreover, high-density materials, such as endodontic fillings and or posts and dental implants within or outside the FOV may affect image quality on CBCT^12,13^. Therefore, the influence of such materials on the visualization of fine anatomical structures requires further investigation.

Optimized acquisition protocols should be selected when a CBCT examination is warranted^8,14,15^. In an ideal scenario, visualization of endodontic structures on CBCT should be performed on images acquired by high-resolution scanning protocols without the presence of metal near to the region of interest^8^. However, in clinical practice, this is not a common condition. Therefore, the present study evaluated 10 CBCT devices with 3 scanning protocols in terms of image quality for detection of cracks and fine endodontic structures under 3 metallic artefact conditions. The null hypothesis was that scanning protocols and the presence of metal does not influence the visualization of root cracks and fine endodontic structures.

## Material and methods

### Ethical aspects

All the methods were designed and approved according to the regulations of the Belgian national council for bioethics research committee (protocol number NH019 2019-09-03) and the Helsinki Declaration. A dried human skull and mandible with full dentition was used in the present study^14,16^.

### Phantom and artefact conditions

**Figure 1:**
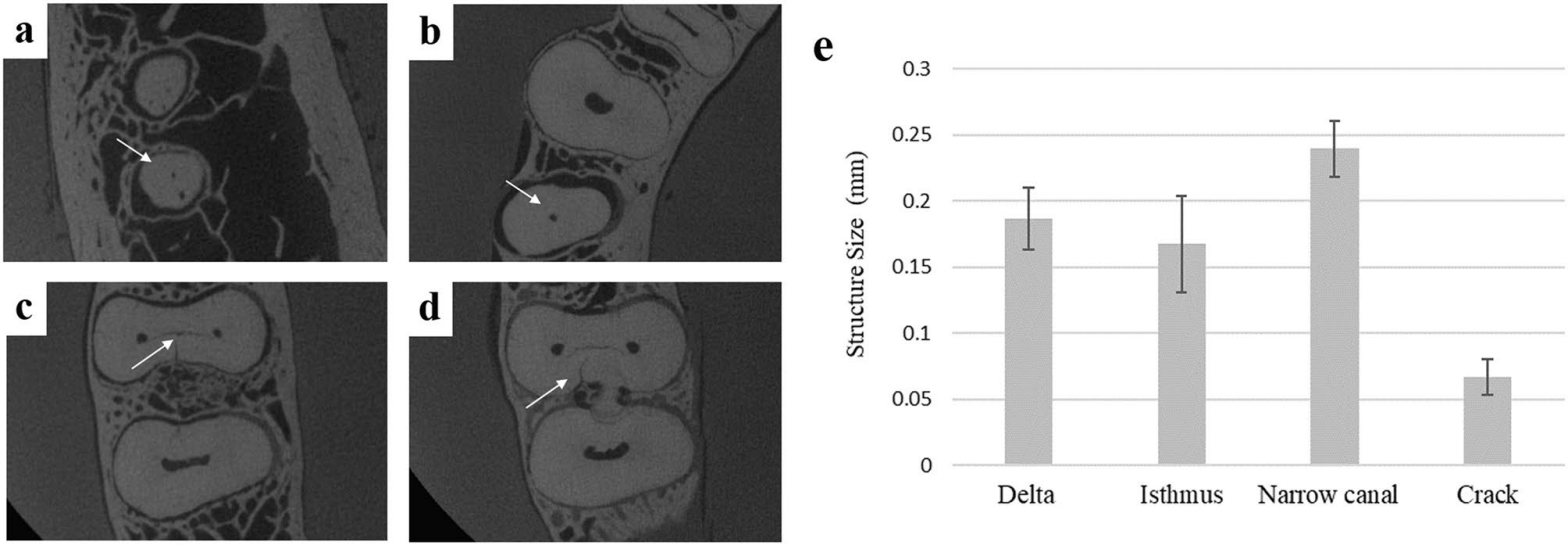
Representative images obtained from industrial CT showing (**a**) delta ramifications, (**b**) narrow canal, (**c**) isthmus and (**d**) crack used as reference images, and (**e**) bar chart showing the means and standard deviations of the size of these structures.

**Figure 2:**
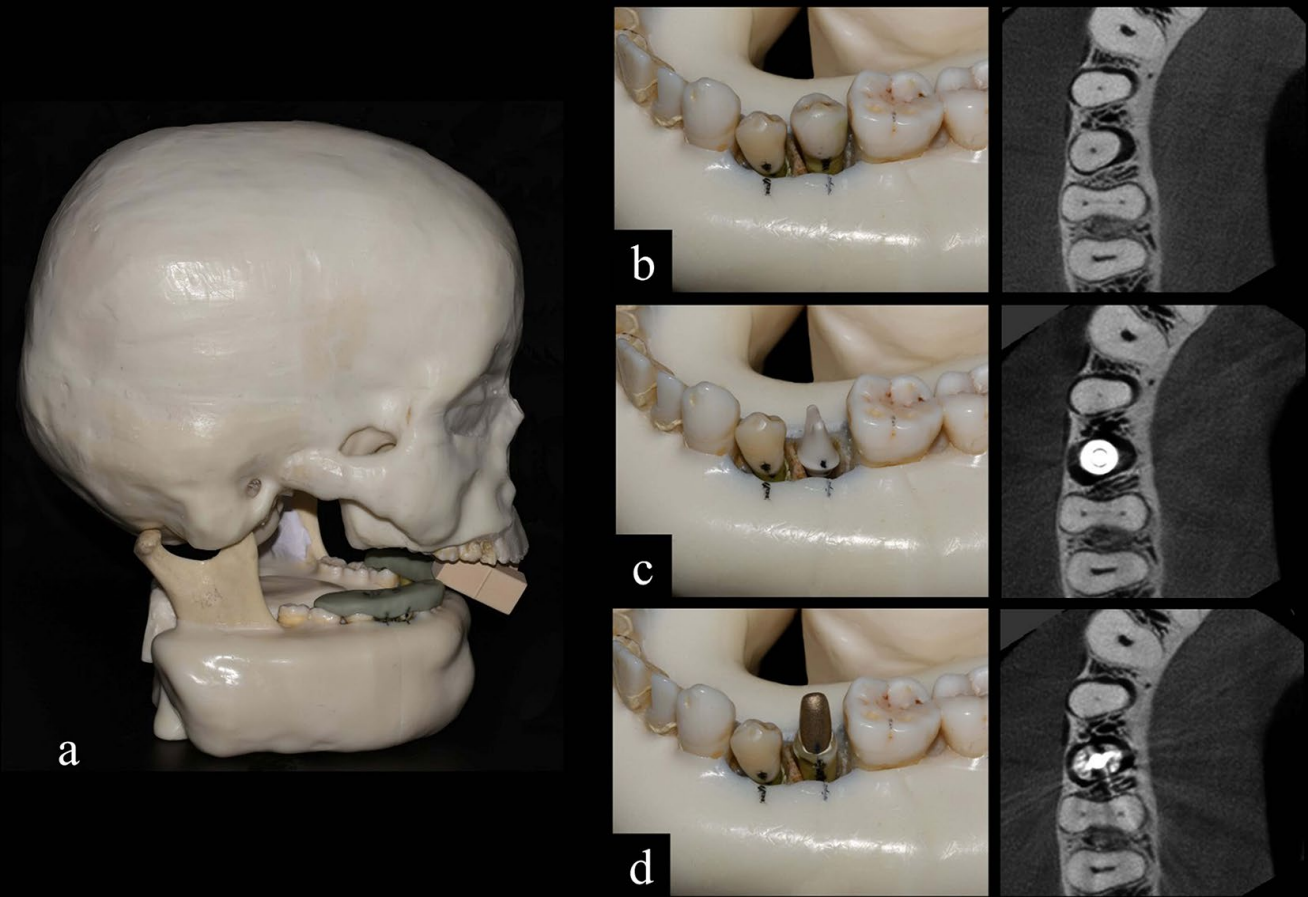
Anthropomorphic phantom of a dry human skull with mandible covered with mix-D (**a**) and representation of the region of interest considering the three conditions evaluated: metal-free (**b**), implant (**c**) and endo (**d**).

An anthropomorphic phantom composed of a dry human skull and a mandible was covered with mix-D material to simulate soft tissue attenuation^14^. Additionally, a mix-D tongue was positioned in the corresponding space of the phantom. A reference industrial image confirmed the presence of a narrow canal in the right mandibular first premolar, and cracks, isthmus, and apical delta in the mandibular first molar (Fig. 1). To assess the influence of metal on the visualization of the aforementioned structures and cracks, three clinical conditions were created (Fig. 2): (1) Metal-free (Fig. 2b); (2) ‘Endo’: with an endodontically treated second mandibular premolar containing a gold alloy metallic post made in-house. The root canals were prepared with Reciproc R25 file (VDW, Munich, Germany) and filled by lateral compaction using AH Plus sealer (Dentsply DeTrey GmbH, Konstanz, Germany) (Fig. 2c); and (3) Implant: the same phantom with a titanium implant with 10 mm length and a platform with 3.5 mm diameter, model Tapered PMC NP (Nobel Biocare, Zurich, Switzerland) placed in the right mandibular premolar region (Fig. 2d). Moreover, silicone guides made with Zetaplus silicone (Zhermack SpA, Badia Polesine, Italy) were used to reproduce the positioning of the teeth for image acquisition.

### CBCT image acquisitions

Ten CBCT devices were selected based on their potential use for endodontic diagnosis, besides their widespread availability worldwide. Subsequently, all conditions were scanned using three protocols (medium FOV standard resolution, small FOV standard resolution and small FOV high resolution) on each of the devices. Table 1 shows all CBCT devices, codes (A to J), exposure parameters and their corresponding dose-area product provided by the manufacturers. The study criteria for the protocols selection were based on the comparison of high-resolution protocols, suggested by the current literature for endodontic assessment, with two others protocols: medium FOV standard resolution, selected in the daily practice for complete mandibular assessment, and small FOV standard resolution. The last one was selected in order to assess the influence of FOV or resolution while keeping the other variables constant. Although some of the CBCT devices tested had an optional metal artifact reduction algorithm, this tool was not used in the present study to avoid confounding variables.

**Table 1:** Detailed description of the CBCT devices, manufacturers, codes, and scanning protocols used in the present study.

CBCT device	Code	Manufacturer	Small FOV high resolution					Small FOV standard resolution					Medium FOV standard resolution				
			FOV (cm)	kV	mA	Voxel (mm)	DAP (mGycm^2^)	FOV (cm)	kV	mA	Voxel (mm)	DAP (mGycm^2^)	FOV (cm)	kV	mA	Voxel (mm)	DAP (mGycm^2^)
3D Accuitomo 170	A	J. Morita, Kyoto, Japan	4 × 4	90	5	0.08	706	4 × 4	90	5	0.125	402	8 × 8	90	5	0.125	2310
OP 3D Pro	B	Instrumentarium, Tuusula, Finland	5 × 5	90	8.6	0.085	755	5 × 5	90	7.1	0.125	452	8 × 8	90	7.1	0.2	899
Veraview X800	C	J. Morita, Kyoto, Japan	4 × 4	100	7.9	0.08	589	4 × 4	101	8	0.125	596	8 × 8	100	8	0.125	2112
X1	D	3Shape, Copenhagen, Denmark	4 × 4	90	12	0.1	309	4 × 4	90	12	0.15	231	8 × 8	90	12	0.15	773
CS 9300	E	Carestream, Rochester, NY, USA	5 × 5	90	4	0.09	572	5 × 5	90	4	0.2	572	7.7 × 7.7	90	4	0.18	455
NewTom VGi evo	F	Cefla Dental Group, Imola, Italy	5 × 5	110	3	0.1	148	5 × 5	110	3	0.125	148	8.2 × 8.2	110	3	0.125	329
Veraviewepocs	G	J. Morita, Kyoto, Japan	–	–	–	–	–	4 × 4	90	8	0.125	525	8 × 8	90	8	0.125	1780
3DR100	H	Dentsply Sirona, Charlotte, NC, USA	5 × 5	85	6	0.079	421	5 × 5	85	10	0.16	214	8.2 × 8.2	85	6	0.16	657
Orthophos SL 3D	I	Instrumentarium, Tuusula, Finland	5 × 5	95	4	0.08	1165	5 × 5	90	4	0.125	582	8 × 8	94	2.8	0.2	940
OP 3D	J	Planmeca, Helsinki, Finland	6 × 6	96	12	0.075	1155	6 × 6	96	7.1	0.15	657	10 × 10	96	7.1	0.15	668

*FOV* field of view, *DAP* dose-area product indicated by the manufacturer.

### Reference image acquisition

The mandible was scanned on a Nikon XT H 225 industrial computed tomography (CT) scanner (Nikon Metrology Inc, Brighton, MI, USA) using the following parameters: 200 kV, 350 µA, 2.5 mm Cu filtration, 8 × 8 cm FOV and 40 µm voxel size. In addition to being used to accurately detect and measure the size of the structures and cracks evaluated, the industrial CT scan was used as a reference to register all CBCT images. For each investigated structure, five different representative slices were selected. For narrow canals and apical delta, the diameter of the canals was measured and for root cracks and isthmus, the width was determined by the arithmetic mean value of five measurements along their length (Fig. 1). All measurements were made by an independent examiner using CTAn software (v.1.14.4; Bruker-micro-CT, Kontich, Belgium). Figure 1 shows the average size for each structure: 0.23 mm for narrow canals, 0.18 mm for apical delta, 0.07 mm for root cracks, and 0.16 mm for isthmus.

### Image registration

The evaluations in the present study were based on comparisons between a set of images within each CBCT device and their respective industrial CT reference image, considering all the different conditions and protocols. Therefore, mutual information-based registration^17^ was performed in Amira 2019 software (Thermo Fisher Scientific, Waltham, MA, USA) using the industrial CT scan as a reference image. The aforementioned registration method allowed the selection of the same slice and consequently ensured the assessment of the same region of interest on all CBCT devices. One hundred and eighty slices per CBCT device were selected to evaluate all diagnostic tasks, comprising five slices for each of the 4 tasks for the three scanning protocols and three artefact conditions.

### Subjective image quality assessment

**Figure 3:**
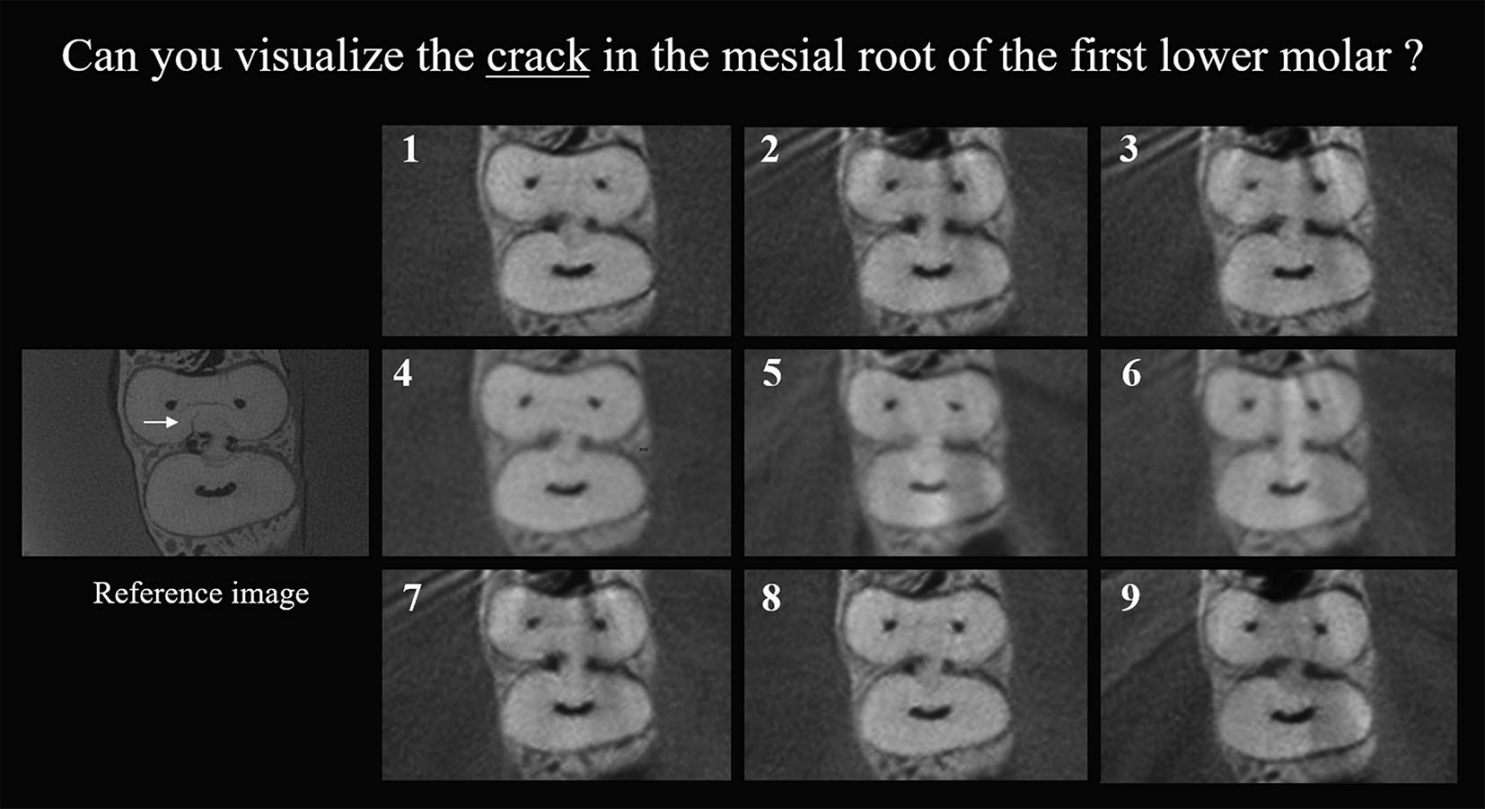
Set of images used for crack assessment containing a reference image from industrial CT and numbered images of X1 CBCT device for all conditions and scanning protocols.

A set of unique images containing fine endodontic structures in other regions of the phantom was used to calibrate two oral radiologists to the study method. After the calibration process the radiologists evaluated each diagnostic task separately. For each device and diagnostic task, there were a set of nine unseen images containing all scanning protocols and artifact conditions randomly displayed on the same computer screen along with the correspondent reference image (Fig. 3). Observers were aware of the appearance and location of the structures of interest and cracks in the reference image, but blinded to the respective CBCT device, protocols and presence of metal. Due to the difference in histogram distribution of each CBCT device, the brightness and contrast of the images were pre-adjusted by an experienced operator to provide images suitable for evaluation of each device. Afterwards, the image brightness, contrast and magnification could not be adjusted by the observers. Both experienced observers assessed the images in consensus by comparison to the reference image and classification into one of the following three categories: 1—appropriate for visualization of the specific structure; 2—acceptable for visualization of the specific structure (the observer could distinguish the structure, however, it was not as clear as in the reference image); 3—inappropriate for visualization of the specific structure (the observer could not distinguish the structure of interest). Calibration and evaluation sessions were performed in a low-light environment using a 61.2 cm MD Barco MDRC-2221 flat-screen monitor (Barco, Kortrijk, Belgium). After 30 days, 20% of the images were re-evaluated to assess intra-observer agreement.

### Statistical analysis

Statistical modeling and analysis were performed using GraphPad Prism 5 (GraphPad Software Inc, La Jolla, CA, USA). Fleiss kappa statistics was used to calculate intra-observer agreement for each CBCT device separately. Kruskal–Wallis and Dunn`s tests were used to compare the different conditions and scanning protocols of each CBCT device with the reference image. A significance level of 0.05 was used for the analysis.

## Results

Kappa values indicate high intra-observer agreement (> 0.81 for all CBCT devices). Figure 1 shows representative industrial CT images of the accessed endodontic tasks used as reference in the present study.

**Figure 4:**
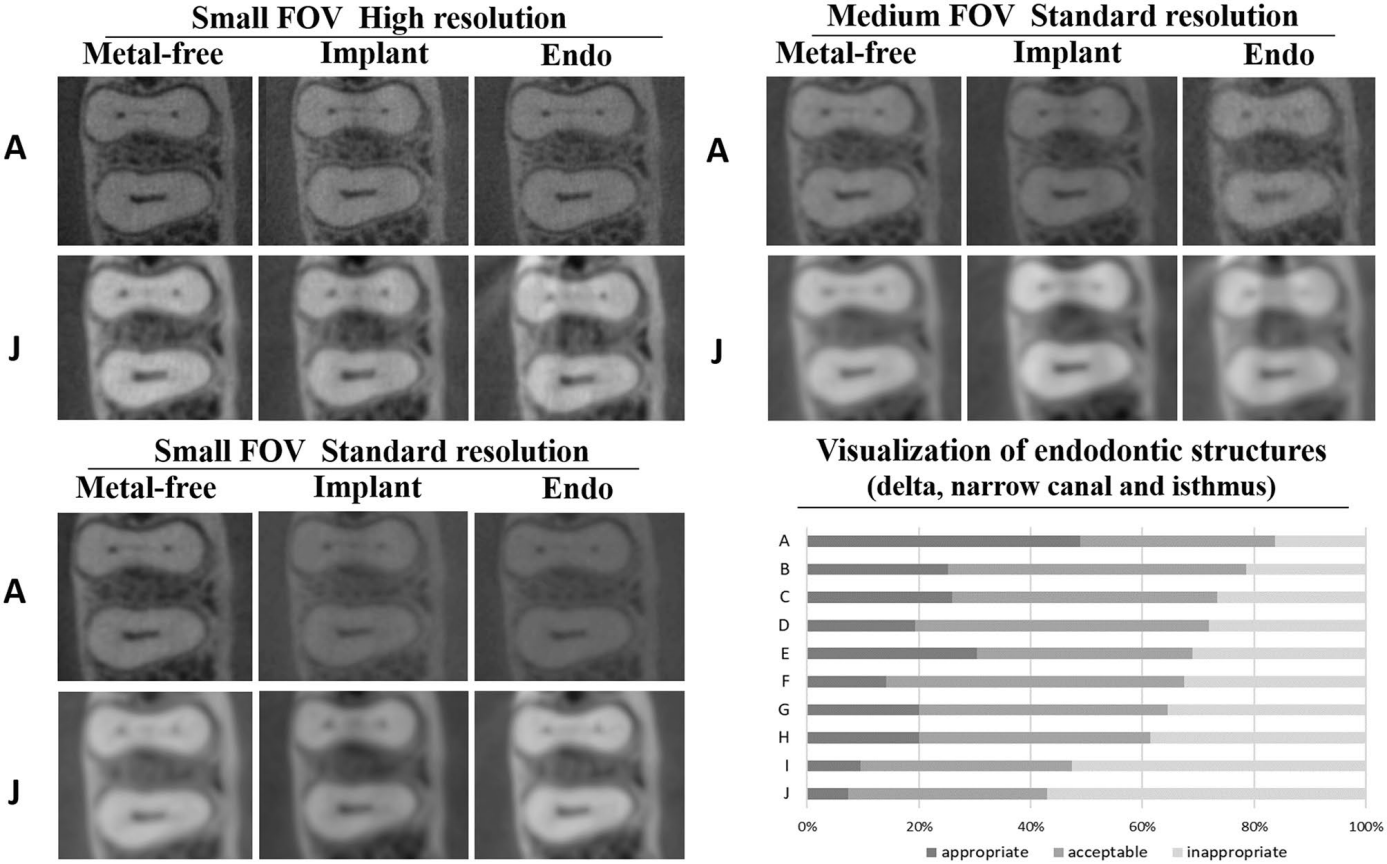
Representative images of isthmus from the devices with the most (A) and least (J) appropriate images for visualization of fine endodontic structures; and stacked bar graph showing the percentage of appropriate, acceptable or inappropriate grades achieved for each CBCT device, taking into account all conditions and the scanning protocol.

**Figure 5:**
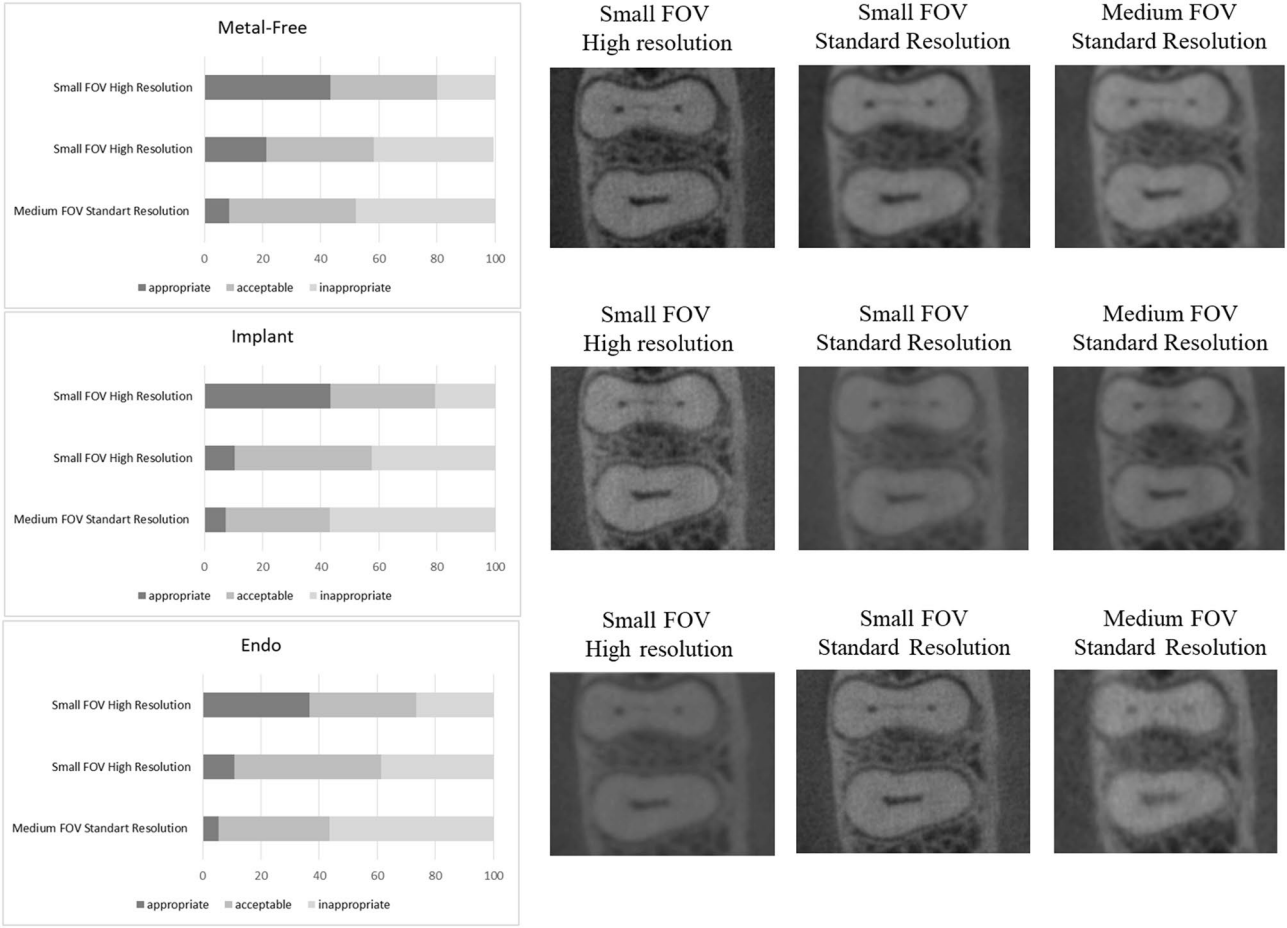
Stacked bar graphs showing the percentage of appropriate, acceptable or inappropriate scores taking into account all CBCT devices for the three scanning protocols and representative images of isthmus from device A.

This figure also contains a bar chart indicating mean values and standard deviations of the size of narrow canal, isthmus, apical delta, and root cracks. A significantly lower size was observed for root cracks when compared to the other evaluated structures (P < 0.05). Figure 4 shows the percentage of images classified as appropriate, acceptable, or inappropriate for each CBCT device, considering all scanning protocols and the presence/absence of metal. Additionally, Fig. 4 shows a set of images from device A which yielded better results (highest percentage of images classified as appropriate) and device J which performed worst (lowest percentage of images classified as appropriate. Figure 5 shows the percentage of appropriate, acceptable or inappropriate scores for all CBCT devices, comparing the influence of the scanning protocol in all conditions. This figure highlights the significantly better visualization of fine endodontic structures in small FOV high resolution images regardless of the CBCT device.

**Figure 6:**
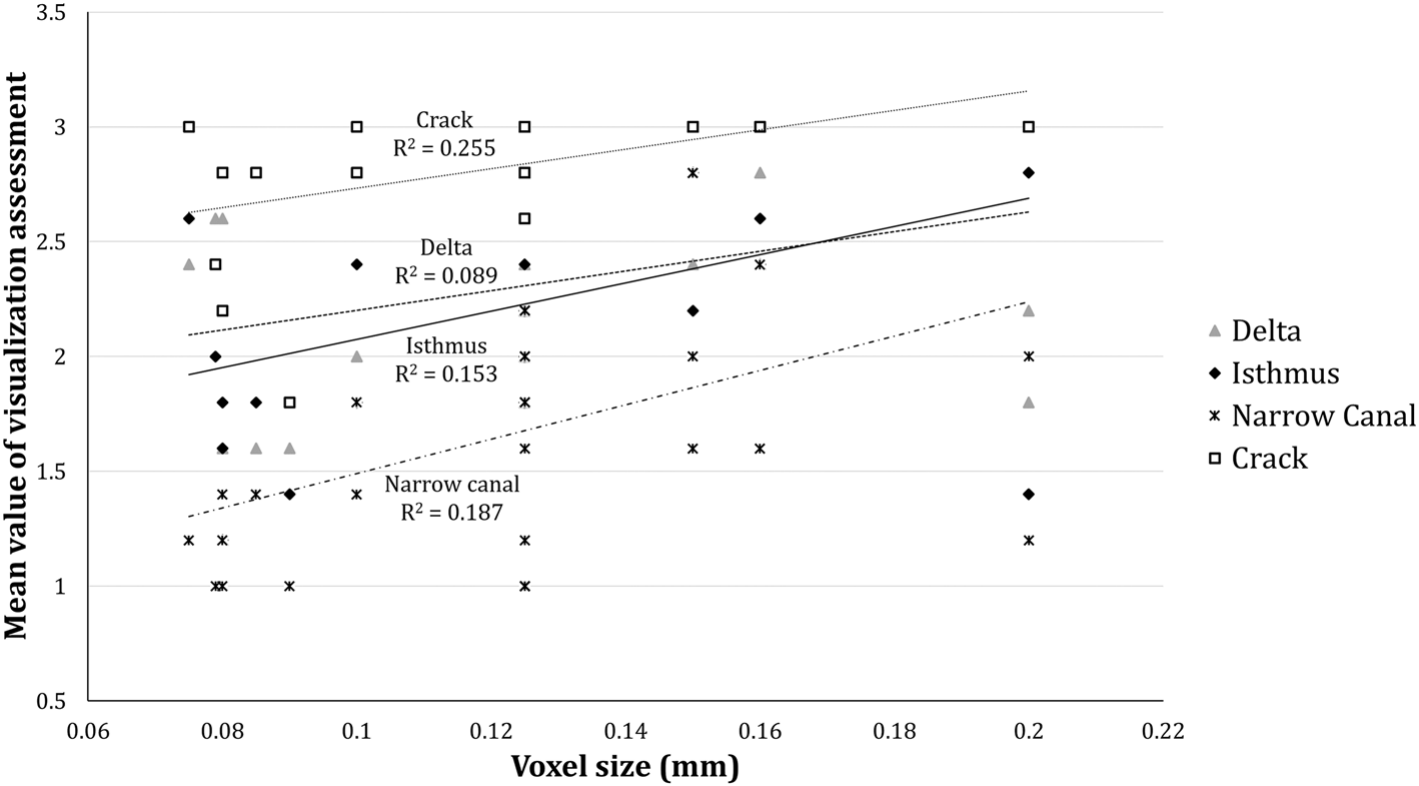
Relation between voxel size and mean assessment grade.

Figure 6 shows the relation between the voxel size and the mean classification score for all scans and tasks. For most devices, there was a significant decrease in the visualization of smaller endodontic structures. A slight correlation was found between the voxel size and classification score for each structure, with the most consistent relation being found for cracks (R^2^ = 0.255), followed by narrow canal (R^2^ = 0.187), isthmus (R^2^ = 0.153) and delta (R^2^ = 0.089).

Tables 2 and 3 show the mean classifications achieved by each scanning protocols (small FOV standard and high resolution and medium FOV standard resolution) and conditions (metal-free, implant and endo) of all CBCT devices and tasks. Moreover, the tables demonstrate multiple comparison tests of each scanning protocol and condition with the reference images.

**Table 2:** Mean, mode and median (med.) values of visualization assessment of root cracks (1—appropriate, 2—acceptable and 3—inappropriate) considering scanning protocols and conditions.

CBCT	Control	Small FOV high resolution									Small FOV standard resolution									Medium FOV standard resolution								
		Metal-free			Implant			Endo			Metal-free			Implant			Endo			Metal-free			Implant			Endo		
		Mean	Mode	Med	Mean	Mode	Med	Mean	Mode	Med	Mean	Mode	Med	Mean	Mode	Med	Mean	Mode	Med	Mean	Mode	Med	Mean	Mode	Med	Mean	Mode	Med
A	1.0	2.2*	2*	2*	2.8	3	3	2.8	3	3	2.8	3	3	3.0	3	3	3.0	3	3	3.0	3	3	3.0	3	3	3.0	3	3
B	1.0	2.8	3	3	3.0	3	3	3.0	3	3	2.8	3	3	3.0	3	3	3.0	3	3	2.6	3	3	3.0	3	3	3.0	3	3
C	1.0	2.8	3	3	2.6	3	3	3.0	3	3	2.8	3	3	3.0	3	3	3.0	3	3	3.0	3	3	3.0	3	3	3.0	3	3
D	1.0	2.8	3	3	3.0	3	3	3.0	3	3	3.0	3	3	3.0	3	3	3.0	3	3	3.0	3	3	3.0	3	3	3.0	3	3
E	1.0	2.8	3	3	2.8	3	3	3.0	3	3	3.0	3	3	3.0	3	3	3.0	3	3	3.0	3	3	3.0	3	3	3.0	3	3
F	1.0	3.0	3	3	3.0	3	3	3.0	3	3	3.0	3	3	3.0	3	3	3.0	3	3	3.0	3	3	3.0	3	3	3.0	3	3
G	1.0	–	–	–	–	–	–	–	–	–	2.8	3	3	3.0	3	3	3.0	3	3	3.0	3	3	3.0	3	3	3.0	3	3
H	1.0	2.4*	2*	2*	3.0	3	3	3.0	3	3	3.0	3	3	3.0	3	3	2.8	3	3	2.8	3	3	3.0	3	3	3.0	3	3
I	1.0	2.8	3	3	3.0	3	3	2.8	3	3	3.0	3	3	3.0	3	3	3.0	3	3	3.0	3	3	3.0	3	3	3.0	3	3
J	1.0	3.0	3	3	3.0	3	3	3.0	3	33	3.0	3	3	3.0	3	3	3.0	3	3	3.0	3	3	3.0	3	3	3.0	3	3

*Statistical similarity with the reference industrial CT scan, which is shown as ‘Control’ (1.0—appropriate for visualizing the root crack).

**Table 3:** Mean, mode and median (med.) values of visualization assessment of fine endodontic anatomical structures (1—appropriate, 2—acceptable and 3—inappropriate) considering scanning protocols and conditions.

CBCT	Control	Small FOV high resolution									Small FOV standard resolution									Medium FOV standard resolution								
		Metal-free			Implant			Endo			Metal-free			Implant			Endo			Metal-free			Implant			Endo		
		Mean	Mode	Med	Mean	Mode	Med	Mean	Mode	Med	Mean	Mode	Med	Mean	Mode	Med	Mean	Mode	Med	Mean	Mode	Med	Mean	Mode	Med	Mean	Mode	Med
A	1.0	1.1*	1*	1*	1.3*	1*	1*	1.5*	1*	1*	1.6*	2*	1*	1.9	2	1	2.0	2	2	1.8*	2*	1*	1.7*	2*	1*	1.9	2	1
B	1.0	1.6*	1*	2*	1.7*	1*	2*	1.9	2*	2*	1.8*	1*	2*	2.1	2	2	1.9	2	2	2.2	2	2	2.3	2	2	2.3	2	2
C	1.0	1.5*	2*	2*	1.6*	1*	1*	2.1	2	2	1.8	2	2	1.8*	2	2	2.1	2	2	2.3	3	2	2.4	3	3*	2.5	3	3*
D	1.0	1.6*	2*	2*	1.6*	2*	2	1.6*	1*	1*	2.2	1	2	1.9	2	2	2.3	2	2	2.3	2	2	2.5	2	2	2.6	3	3
E	1.0	1.3*	1*	1*	1.3*	1*	1*	1.5*	1*	1*	1.5*	1*	1*	2.0	2	2	2.2	2	2	2.7	3	3	2.8	3	3	2.7	3	3
F	1.0	1.6*	2*	2*	1.8*	2*	2*	2.1*	1*	2*	2.3*	2*	2*	2.5	3	3	2.3	2	2	2.3	2	2	2.4	2	2	2.4	2	2
G	1.0	–	–	–	–	–	–	–	–	–	1.6*	2*	2*	2.3	2*	2*	1.9	1	2*	2.3	3	2*	2.6	3	3	2.0	2	2*
H	1.0	1.5*	1*	1*	1.5*	1*	1*	1.6*	1*	1*	2.5	2	2	2.7	2	3*	2.7	3	3*	2.3	2	2	2.4	2	2	2.4	2	2
I	1.0	1.9*	1*	2*	1.9*	2*	2*	2.3	2	2*	2.9	3	3	2.2	2	2	2.1	2	2	2.9	3	3	2.8	3	3	2.8	3	3
J	1.0	2.1	2	2	2.3	2	2	2.1	2	2	2.9	3	3	2.9	3	3	2.5	3	3	2.3	2	2	2.5	3	3	2.8	3	3

*Statistical similarity with the reference industrial CT scan, which is shown as ‘Control’ (1.0—appropriate for visualizing the anatomical structures).

Table 2 indicates that only devices A and H presented similar performance to the reference for visualizing root cracks in the metal-free condition scanned with a small-FOV high-resolution protocol.

Scanning protocols influenced the visualization of endodontics structures (narrow canal, delta apical and isthmus), with small-FOV high-resolution protocols performing better than the others (p < 0.05), as seen in Table 3. The presence of a metallic post decreased the ability to visualize the structures even for high resolution protocols (p < 0.05). However, similar behavior was not observed when the implant was present (p > 0.05). In the absence of metal, the small FOV standard resolution protocols from devices A, B, C, E, and G displayed similar visualization to the reference image. In the presence of implants, only device C remained similar to the reference (p > 0.05). For medium FOV standard resolution, only device A did not differ from the reference in the presence of metallic structures. Device J was not able to perform similarly to the reference image for any of the protocols and conditions (p < 0.05). Detailed categorical data obtained in this study can be seen in the supplementary information section (Supplementary information).

## Discussion

This was the first study to assess the influence of scanning protocols and metallic materials on the visualization of root cracks and fine endodontic anatomical structures using ten different CBCT devices. These devices were selected considering their widespread availability worldwide and their use for endodontic diagnoses and treatment planning. Furthermore, out of all available CBCT devices^11^ the selected devices represent the large variability in terms of CBCT technology currently available.

The null hypothesis was rejected, as all tasks were influenced by scanning protocols and presence of metallic materials. The small-FOV high-resolution protocols provided images with appropriate or acceptable visualization capability of endodontics structures (narrow canal, delta apical and isthmus) for all devices evaluated, except for device J. This result confirmed that most CBCT images acquired with limited FOV and high-resolution protocols are preferable for fine endodontic structure visualization^18^. However, it is noteworthy that some devices have provided images similar to the reference even when small FOV standard resolution was selected. Additionally, device A was similar to the reference regardless of the protocol. The varying performance for scanning protocols from different CBCT devices may be explained by differences in terms of other technical features amongst CBCT devices, such as focal spot size, detector pixel size, number of projections, and reconstruction filters^19^.

High-density materials such as filling materials, gutta-percha and metallic posts may cause device-specific artifacts, reducing CBCT image quality^20–22^. The present results highlighted a decrease in the ability to visualize small endodontic structures in images with metallic posts within the FOV. Previous investigations observed that artifacts generated from metallic posts have high impact on image quality^23,24^. Interestingly, the presence of an implant did not influence the behavior of most devices and scanning protocols. These results may be explained by differences in terms of atomic number of the gold metallic post (Z = 79) and titanium implant (Z = 22), since materials with higher atomic numbers are associated with more artifact expression in CBCT^25^. Although several studies have shown the impact of artifacts in regions adjacent to dental implants on CBCT images^12,26,27^, few of them evaluated their influence on the visualization of structures in adjacent teeth, and certainly none of them in a series of CBCTs with a standardized condition as in the present study.

Concerning crack visualization, only two devices performed similar to the reference image, and only for a specific condition and scanning protocol. While the relatively low average width (0.07 mm) of cracks complicated an accurate measurement due to the finite voxel size of the reference scans, cracks could be easily detected on the reference images owing to the superior high-contrast resolution as compared to clinical CBCT scanners. The partial volume effect may explain the difficulty of detecting structures, which are smaller than the voxel size^28^. This effect occurs when a single voxel represents two or more structures, precluding their proper identification^29^. Figure 6 shows that for most devices, there was a significant decrease in the visualization of endodontic structures with decreasing structure size. A narrow canal (mean size of 0.23 mm) showed the best overall visualization while cracks (mean size of 0.067 mm) were assessed as having the worst visualization. Furthermore, a slight correlation between the voxel size and the mean score for a given scan condition was found. Of course, voxel size is one of many factors affecting detectability limits in tomographic images. The actual spatial resolution depends on the focal spot size, mechanical (in)stability of the scanner, detector pixel size and binning, number of projections, and reconstruction filter. Furthermore, the visualization of a structure is dependent on its contrast, and thus, the contrast resolution of the image. In addition to the limitation in overall sharpness, the presence of high-density materials generates artifacts that mimic or overlap fracture lines^23,30^. Other studies have also discussed the aforementioned limitations for detecting cracks on CBCT images and their size is undoubtedly one of the most crucial factors^9,31^. However, when diagnosing root cracks and fractures, other factors should be taken into consideration, such as clinical signs and symptoms, and the pattern of bone loss on images^13^. Furthermore, the exposure parameters, detector technology, and reconstruction algorithms should also be considered for diagnostic purposes^5^. It is important to keep track of the constant evolution of technology and investigate how this may lead to improve diagnostic efficacy.

Mean values were used in Tables 2 and 3; while the use of mean for ordinal data is debatable, we considered the values of the three scores on the scale used by the observers equidistant from a semantic point of view. Thus, mean values depict small to moderate changes in scoring between scan conditions (which would typically not be reflected by a median or mode). As the present ex-vivo study aimed to compare a set of registered images with a similar reference image, it is obvious that in vivo CBCT imaging in human could not be performed. Thus, factors such as patient motion and patient variability could not be assessed^32,33^. When evaluating a set of images, observers were aware of the presence of a specific anatomic structure. Hence, the aim was to assess CBCT image quality in relation to the reference image, rather than determine diagnostic sensitivity and specificity. The use of an anthropomorphic phantom was the only way to evaluate the same scanned structure by selecting various protocols on ten CBCT devices. Image registration using a reference image allowed standardizing the regions of interest, consequently improving the reproducibility of the study^34^. The anthropomorphic phantom was composed of a complete dried human skull and a mandible covered with a soft tissue substitute material^14^. This dedicated material, called mix-D, provides similar attenuation to soft tissues. In addition, the endodontic challenging tasks evaluated were naturally pre-existing, avoiding the limitation related to its artificial creation.

The present study highlighted the influence of different scanning protocols and the presence of metal structures within the FOV on the visualization of root cracks and challenging endodontic tasks. Furthermore, the results showed that this influence is device-dependent. As long as there are no high-density objects in the scanned region, several CBCT machines may be suitable. Yet, once high-density materials are present, fine endodontic diagnosis may be compromised in many CBCT machines. For small root cracks (average diameter of 0.07 mm), CBCT diagnosis in the presence of metallic structures becomes extremely challenging, even though a comprehensive dental history and specific clinical findings might guide the diagnostic process^19,35^.

## Conclusions

The ability of CBCT images to detect root cracks is restricted to certain CBCT devices and protocols in absence of metal. Once metal artefacts are present, crack detection becomes unlikely. Visualization of fine endodontic structures is also CBCT-device dependent with a better capacity observed for small-FOV high-resolution protocols as long as there are no high-density objects adjacent to the region of interest (Supplementary information).

## Supplementary Information

Supplementary Information.

## Data Availability

The datasets used and analyzed during the current study are available from the corresponding author on reasonable request.
